# Culture-bound syndromes in migratory contexts: the case of Bolivian immigrants

**DOI:** 10.1590/1518-8345.1982.2915

**Published:** 2017-07-10

**Authors:** María Teresa Roldán-Chicano, José Fernández-Rufete, César Hueso-Montoro, María del Mar García-López, Javier Rodríguez-Tello, María Dolores Flores-Bienert

**Affiliations:** 1 PhD, RN, Hospital General Universitario Santa Lucía, Servicio Murciano de Salud, Cartagena, Murcia, Spain.; 2 PhD, Researcher, Murcia, Spain.; 3 PhD, Full Professor, Universidad de Granada, Granada, Spain.; 4 MSc, RN, Centro de Salud Barrio Peral Servicio Murciano de Salud, Cartagena, Murcia, Spain.; 5 MSc, RN, Hospital General Universitario Santa Lucía, Servicio Murciano de Salud, Cartagena, Murcia, Spain.; 6 PhD, Full Professor, Universidad de Murcia, Murcia, Spain.

**Keywords:** Cultural Characteristics, Immigration, Cultural Competency, Traditional Medicine, Bolivia, Transcultural Nursing

## Abstract

**Objective:**

to describe the culture-bound syndromes maintained by Bolivian immigrants in the new migratory context and analyze the care processes of these health problems.

**Method:**

qualitative research with an ethnographic methodological approach. Sample: 27 Bolivian immigrants. In-depth interviews and participatory observation were the strategies used for data collection. Data were classified and categorized into logical schemes manually and using the ATLAS-ti program v.5.

**Results:**

susto, “wayras”, amartelo, pasmo de sol, pasmo de luna and pasmo de sereno are some of the folk illnesses that affect the Bolivian immigrants and that they have to treat in the new migratory context.

**Conclusions:**

in the new environment, the group under study preserves culture-bound syndromes that are common in their country of origin. The care strategies used for these health problems are adapted to the resources of the new context and based on interactions with the domestic environment, biomedicine and traditional medicine. It was observed the need for the health professionals to realize that the efficacy of certain therapies occurs within the scope of cultural beliefs and not in that of the scientific evidence.

## Introduction

Biomedicine builds abiological, ahistorical and acultural diagnoses based on the language of the “empirical”. This “language of the concrete” gives them a scientific and objective basis, which confers their universal character. However, when these diseases are built as “culture-specific syndromes”^1^ (*Culture-Bound Syndromes*), their complex etiology, description and treatment prevent them from having a “universal character”, which at first, makes them specifically limited to certain geographic areas and cultural spheres. 

A culture-specific syndrome arises “when the members of a cultural group or community, by mutual agreement, identify a particular pattern of signs and symptoms, to which they attribute a certain causality, meaning and therapeutics, so that they become entities strongly influenced by the cultural context from which they arise”. In such cases, it is common the use of procedures of symbolic efficiency for the recovery of the patient^1^.

Research in the field of folk medicine has made it possible to group a wide variety of cultural diseases: *hwa-byungin* in Korea^2^, *hikikomori* in Japan^3^, *empacho* in Chile^4^, *evil eye* in the Mediterranean countries, etc. Mental Health and Transcultural Psychiatry is the field that has dedicated increasing attention to these health conditions and has devoted its best efforts in their classification and categorization^6^ ^-^ ^7^.

For some authors, culture bound/specific syndromes cannot be dissociated from their cultural context^8^ because their etiology brings together and symbolizes fields of meaning and ground rules of behavior in society. The patient needs to believe in this reality and become a member of a society that also believes in it. Therefore, illness is crystallized into a worldview and the recognition of its reality depends on the degree to which a particular cultural universe is shared. However, what happens when this worldview is not shared?

The social dimension of the disease, or the process of socialization of health problems^9^ have, in the case of immigrants, a special complexity since the entrance into a new context represents a great change^10^. This disconnects the immigrant from the cultural environment where the semantic networks and the socially shared definitions of what the disease is or is not emerge. In this environment, where acculturation seems to be an unequivocal destination, how do immigrants deals with these diseases? How does the immigrant community deal with culture-bound illness in contexts where biomedicine exercises its hegemony?

The ability to emigrate is one of the distinctive features of our species and is the basis of our evolutionary success, but the intense stress levels in the migration process may compromise the capacity of immigrants to adapt and their health^11^ ^-^ ^12^. Regarding the culture-bound syndromes, they have been widely studied in the countries of origin, but there are few studies addressing them in the new migratory context^13^ ^-^ ^14^

The studied group, Bolivian immigrants, are characterized by a great cultural diversity, since Bolivia is one of the countries with greatest ethnic diversity (36 peoples form the ethnic map of Bolivia)^15^. This great diversity creates, depending on the cultural group in which we will focus our observations, a large repertoire of diseases with a cultural origin. This study aims to find out whether the folk illnesses of the Bolivian immigrants coming from the *Quechua*, *Aymara* and *Guarany*/*Guarayo* groups, as well as those who do not self-identify with any ethnic group, are maintained in the new migratory context in Spain. The objective of this study is to describe the health-disease-care processes related to these culture-bound syndromes.

## Method

Qualitative research project with a “particularist” or “focused” ethnographic methodological approach, carried out in the region of Murcia, in the Southeast of Spain. 

Participants were Bolivian immigrants living in the Region of Murcia. The inclusion criteria to participate in the study were: length of residence in Spain of at least two years, minimum age of 18 years, consent to participate in the study.

This research is based on some theoretical assumptions of Anthropology, such as the “self-care”^16^, in which the individual interprets its illnesses and makes an articulated use of different care strategies. The study of these concepts requires two essential strategies for data collection: in-depth interviews and participatory observation. 

During the interviews, in order to limit the topics of interest, a series of broad questions was designed and a first script was defined, which was later remodeled as the theoretical sample profile changed. The interviews ranged in duration from 30 to 60 minutes. The participants’ comments transcribed in this study are part of the interviews, the letter E refers to “Interviewer” and the letter I refers to “Informer”.

In total, 27 interviews were conducted, 16 with women and 11 with men, and they were carried out by the main researcher of the project who has previous experience in the area of qualitative research. The age range of the interviewees varied from 21 to 55 years and the average age was 30 years. Of the interviewees, 19 originally lived in an urban environment and the rest came from a rural environment. Most of the informers came from the Department of Cochabamba, and many of them identified themselves as *Quechua*. This study could have limited its objective to the Andean worldview, however, the interviews and participatory observations carried out with the immigrants from the Eastern part of Bolivia provided a broader and more varied perspective. Of the total number of interviewees, 11 identified themselves as belonging to a specific ethnic group (8 *Quechuas*, 1 *Aymara*, 1 *Guarani* e 1 *Guarayo*), as shown in Table 1.

**Table 1:** Sociodemographic characteristics of informers. Cartagena, Murcia, Spain, 2015

I*D	**Department**	**Urban or rural origin**	**Self-identification with ethnic group**	**Age**	**Sex**
I* 1	**Cochabamba**	**Rural**	**Yes,** *Quechua*	**35**	**M** ^**†**^
I* 2	**Oruro**	**Rural**	**No**	**30**	**M** ^**†**^
I* 3	**Cochabamba**	**Rural**	**No**	**29**	**M** ^**†**^
I* 4	**Cochabamba**	**Urban (Cochabamba)**	**Yes,** *Quechua*	**33**	**M** ^**†**^
I* 5	**Santa Cruz**	**Urban (Santa Cruz)**	**Yes,** *Guaraní*	**27**	**M** ^**†**^
I* 6	**Cochabamba**	**Urban (Cochabamba)**	**No**	**21**	**F** ^**‡**^
I* 7	**Cochabamba**	**Urban (Sacaba)**	**Yes,** *Quechua*	**50**	**F** ^**‡**^
I* 8	**Cochabamba**	**Urban (Sacaba)**	**Yes,** *Quechua*	**55**	**M** ^**†**^
I* 9	**Cochabamba**	**Urban (Sacaba)**	**No**	**30**	**F** ^**‡**^
I* 10	**Cochabamba**	**Urban (Sacaba)**	**Yes,** *Quechua*	**31**	**F** ^**‡**^
I* 11	**Cochabamba**	**Rural (Punata)**	**No**	**27**	**M** ^**†**^
I* 12	**Cochabamba**	**Rural**	**Yes,** *Quechua*	**37**	**M** ^**†**^
I* 13	**Cochabamba**	**Urban (Cochabamba)**	**No**	**20**	**F** ^**‡**^
I* 14	**Cochabamba**	**Urban (Cochabamba)**	**No**	**22**	**F** ^**‡**^
I* 15	**La Paz**	**Urban (Quime)**	**No**	**20**	**F** ^**‡**^
I* 16	**La Paz**	**Urban (Quime)**	**Yes,** *Aymara*	**20**	**M** ^**†**^
I* 17	**La Paz**	**Urban (Quime)**	**No**	**22**	**F** ^**‡**^
I* 18	**Cochabamba**	**Rural**	**No**	**21**	**F** ^**‡**^
I* 19	**Cochabamba**	**Urban (Sacaba)**	**Yes,** *Quechua*	**24**	**F** ^**‡**^
I* 20	**Santa Cruz**	**Urban (Santa Cruz)**	**No**	**40**	**F** ^**‡**^
I* 21	**Cochabamba**	**Urban (Vinto)**	**Yes,** *Quechua*	**27**	**F** ^**‡**^
I* 22	**Cochabamba**	**Urban (Vinto)**	**No**	**30**	**F** ^**‡**^
I* 23	**Beni**	**Urban (Trinidad)**	**No**	**34**	**M** ^**†**^
I* 24	**Santa Cruz**	**Urban (Santa Cruz)**	**Yes,** *Guarayo*	**35**	**F** ^**‡**^
I* 25	**Santa Cruz**	**Urban (Santa Cruz)**	**No**	**30**	**M** ^**†**^
I* 26	**Cochabamba**	**Urban (Cochabamba)**	**Yes,** *Quechua*	**40**	**F** ^**‡**^
I* 27	**Cochabamba**	**Urban (Cochabamba)**	**Yes,** *Quechua*	**43**	**F** ^**‡**^

*I=Informer; †Male; ‡Female

Participatory observation was carried out in markets, call shops, cyber cafes, grocery stores and public places, such as parks and gardens in different localities. The main researcher maintained close contact with a Bolivian family for a month, which allowed her to gain a deeper understanding of the domestic life and the self-care patterns of the group. This close contact also allowed expanding the relationship with more Bolivian immigrants, making it possible to collect data in other scenarios: other domestic environments (this time, discontinuously), bars, restaurants, discos, etc. It was also possible to watch various events and celebrations of the families (birthdays, baptisms and various celebrations) or community (celebration of the day of the *pacha-mama*). Therefore, “snowball” sampling was the most used method during the research, although at the end of the study, when part of the data had already been analyzed, priority was given to a theoretical sampling whose selection of informers occurred in a more selective way.

Field work (participatory observation and interviews), which was often added to the analysis, was carried out discontinuously from August 2009 to August 2015 and finished when the theoretical saturation was reached and when the information provided by the informers became redundant or unimportant for the ethnographic analysis.

The information collected in the interviews by means of audio recordings was transcribed, contrasted and enriched with the registered field work data during participatory observation. Three researchers organized the data manually and using the program ATLAS-ti v.5, into codes, categories and, later, logical schemes that could explain the culture-bound syndromes of the group under study.

All participants (interviewees and those present during participatory observation) were informed about the profile and interests of the researcher who collected the data and about the characteristics of the research project (how data would be collected, potential risks and benefits of participating and how the information would be kept confidential). With this information, it was ensured that those who gave their consent to participate would have values, interests and preferences compatible with this research project. The information was communicated to the participants in a clear and comprehensive way, and the contact information (telephone and *e-mail*) of the main researcher was provided, if they decided to withdraw from the study. Eight people declined to participate in the survey, most of them arguing lack of time to be interviewed, and none revoked their consent.

Aiming to reinforce the validity of the study, a triangulation was performed between the data collected by the main researcher and two other researchers. A preliminary analysis of the data was shown to 3 of the informers to verify if they shared and approved the descriptions and interpretations on the culture-bound syndromes and the therapeutic procedures for their treatment.

## Results and Discussion

### Susto or fright

According to the concept of human being typical of the cultural and linguistic groups *Quechua* e *Aymara*, from the Andes, a person is composed of a body and a series of psychic elements that are known “*ajayu*”, “essence”, “courage” ou “spirit”^17^.

This category of psychic elements involves a hierarchy. The loss of the main psychic element, “*ajayu*”, involves the death of the individual. On the other hand, if other psychic elements (“essence”, “courage” or “spirit”) that play a secondary role in the vital force of the individual are lost, the individual would suffer from a condition with a very confusing symptomatology^18^, which can be treated using appropriated ritual practices^19^. 

All informers from the Bolivian Andean plateau considered the *fright* as a disease that they themselves or other relatives had suffered from in Bolivia as result of any unexpected occurrence. Some informers reported that they have not suffered from *fright* in Spain because here, in their new way of living, its causal agents had disappeared...

*In Bolivia, I used to have “fright” when my own brothers gave me a scare, or when a dog or a cow scared me ... or even a frog coming out of some rocks or dry leaves ... (...). Here, in Spain, there are no frogs to scare you, and every time I get frightened, I notice red stains in my body and have a very strong headache.* (I 4)

Although adults may also get frightened, children and infants are the most sensitive groups, and therefore, more prone to have a *fright*. In Spain, only two informers reported cases of *fright*, both of them have occurred in children. In one case, the cause of the *fright* was not identified and in the other one, the exposure of the child to a loud noise was the cause.

… *my husband and I did not believe in these things ... but when my daughter was born ... children are sensitive to any loud noise ... a loud noise cause her the fright ...(…)... she cried and cried all night long and she would not stop…my mother said that my daughter was scared ... your daughter is scared ... because she was sleeping and suddenly awakened ... and started to scream ...(…), and as consequence, it was necessary to call her spirit back.* (I 10)

The different ways of healing the “*fright*” in the Bolivian plateau include the intake of medicinal plants^20^ and “to call of the lost soul”, which is made through rituals of offerings (made by *yampiris* or *yatiris*) or through the “white table”, performed by the *kallawayas*, who play other roles in the traditional Andean medicine^19^.

The treatment for the *fright* in Spain acquires a domestic nature, since the impossibility of contacting any *yatiri* or *kallawaya* causes any member of the family group to take on the role of the healer in treating the *fright*. As reported by the informers, to heal the *fright*, it is necessary to “have experience and know how to call the spirit”, making the responsibility of this practice fall on the oldest member of the family. In both cases analyzed, the grandparents of the two children suffering from “*fright*”

*... to call the spirit? ... here it has been done by my aunt for the baby ... his little spirit has been called with the use of incenses ... (...) ... my aunt did it because the baby got a fright ..., and he would not stop crying ..., (...) ... it can be done by someone who has experience ..., someone who knows how to call the spirit, and these kind of things.* (I 9)

To call the lost spirit, it is necessary to wait for the night, burn the incense and, with a worn piece of clothing that has not been washed, claim the return of the soul that “remained in the place where the child got a fright”.

*... after the fright, the spirit remains where the fright happened, so it is necessary to call the spirit at midnight, when people are sleeping ... then you must call the spirit using the little girl’s cap or using a girl’s worn piece of clothing that has not been washed, that the girl had worn ... then someone goes outside and calls the spirit ...(…) incense is used with embers and charcoal ... just as incense is used in the churches, and like this, the spirit is called “Come my little angel, come, come”... and so it is being called back ... and they start getting a good night’s sleep in the very next day, that is ... seeing is believing.* (I 7)

The *fright*, or the physical and psychological symptoms triggered by this syndrome, can also be diagnosed and treated by biomedicine^14^ ^,^ ^17^. In Spain, possibly more often than in Bolivia (due to the impossibility of treating the *fright* through the traditional Andean medicine), the one who has lost his *spirit* may decide to see a doctor to mitigate the symptoms. One of the informers, when analyzing the possibilities to treat the *fright* in Spain, describes the treatment for this health problem using the assumptions of biomedicine.

*... the fright ... is like to have been separated from the spirit at that moment ... and to cure it, well, here there are doctors ... there are more possibilities to see the doctor, and the doctor will give you injections (...) that’s because the person is apathetic, his head hurts ... then they give you injections.* (I 1)

### Machu wayra , limbu wayra : the bad devil’s wind

Sometimes, when someone is sleeping, a *devil’s wind*, a *bad wind*, *machu wayra* or *limbu wayra*, can get the individual and cause him illness. One of the informers suffered from *bad wind* in Spain...

*The bad wind can catch the elderly and children at any night, this is a very serious and bad disease, it hurts like cramps, children start crying and cannot sleep ... (...) the devil’s wind is on the air... (...) ... on the day, nothing happens to you, but three days later or more you get bad, the bad wind gets you and you feel painful from the waist down ... and then you need to go see a healer, a jampiri ... the jampiri heals you because he talks to the devil, says this is a devil’s wind ... when the bad wind gets you, you walk down on the streets with your head down as if you were upset or bored ... you cannot sleep, you feel pain or you have nightmares.* (I 21)

*Has this happened to you in Spain?* (E)

*Yes, I usually cry when I get upset ... I remember my family and that this has already happened to me, the bad wind catched me, my hands started to hurt and I got a cramp and I felt like I could not stay here any longer.* (I 21)

The “*wayra*” are a group of diseases carried by the wind, which in the case of the informer, triggered “refusal”, boredom and neurological symptoms. In view of this health problem, the informer reports that she went to see a doctor, but she realized that he could not solve her problem. Thus, she decided to see a “*Spanish* *yampiri*”, who did not heal like the *jampiris* in her country although he also “prayed”.

The *Spanish jampiri*, as the informer called him, is actually one of the witch doctors in Spain, usually known as traditional healers. Healers claim to have an innate *grace* that allows them to heal different diseases, ranging from evil eye to herpes. Flexibility in their performance spectrum has allowed them to combine a wide array of diseases (and clients) that, although have its origin in the Andean worldview, seem to be susceptible to cures with a ritual nature.

*When the bad wind caught you in Spain, what did you do?* (E)

*Here, there are also jampiris (...) Spanish ones, in Alhama there are some ..., an Ecuadorian friend of mine took me to see one when I was very sick, the doctors did not solve my health problem..., they did not do anything to cure me, I went to the medical center and the doctor I gave me an injection, but it was not enough to calm me down, after that, an Ecuadorian lady told me that it was better to go to see a jampiri. The Spanish jampiri had my back to him and said a prayer ... (...) he has cured me saying a prayer to God and to the Virgin Mary ..., he asked me to tell him my name ..., he asked my name and said a prayer for me ... and said that I could give him a voluntary contribution of €5 or €10 or €20 (...) and gradually I started to feel better, I got cured*. (I 21)

Although the Spanish healer does not invoke or mention any of the protective entities of the Andean worldview (*chullpas*, *pachamama*, *machu wayra* or *limbu wayra*), the symbolic efficacy of his prayer is more similar to the traditional healing strategies of the Andean medicine than those of the biomedicine (which were considered as ineffective by the informer).

The syncretism in the healing rituals of the *kallawayas*, *yatiris* and *jampiris*, in which Christ and saints share the protagonism with the *pachamama* and the ancestral *chullpas*, allows the patient to recognize some protection entities that are part of its worldview in the prayers of the healer of Alhama. This also allows the patient to consider this practice as logical and coherent. The representations invoked by the healer of Alhama in praying to God and to the Virgin seem to have modified the physiological functions of the patient, who was, in his own words, healed by the prayers of the healer.

### Pasmo de sol , pasmo de lua and pasmo de sereno 

The informers from the eastern part of Bolivia have a cultural background that is part of the beliefs and values characteristic of the *guarayos* and *guaranis*, or belong to these communities, which are some of the ethnic groups inhabiting the Department of Beni and Santa Cruz. Bolivian immigrants from these regions describe a health problem called “*pasmo*” that, according to some explanatory models of the disease, it would be considered as an exogenous condition, in which the disease is due to the action of an element (real or symbolic) unrelated to the patient. 

The informers have only described the cases of *pasmo* occurring in Bolívia, however, in Spain, self-care practices have been performed to prevent them.

*I have met people who died from sitting down on the heat ... or eating a warm fruit ... (...) Yes, this is a terrible evil that can kill you ... and overnight, when the dew falls down, you can not eat those wet fruits either, because it’s bad for you, you have to let them dry ... (...) ... the same happens with the moonlight ... because there is pasmo de sol and pasmo de luna…, the moon can also cause you pasmo, the moonlight actually (...) and pasmo is less severe in adults than in babies ... you can not neglect the babies ... because the moonlight may cause them skull fractures and they die ... because the bones in their skull are not yet joined (it points to its head) ...., and if a child is left like that, it dies ... the child cries, cries, cries and then dies ... and it is necessary to heal the child with herbs.* (I 5)

**Figure 1:**
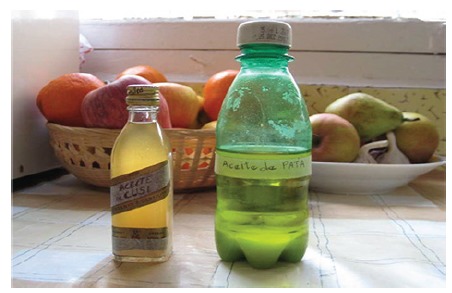
Photograph of “neatfoot oil” (right) and “babassu oil” (left). Cartagena, Murcia, Espanha, 2015

Newborns are especially vulnerable to “*pasmo de luna*” and to “*pasmo de sereno*” because of the weakness of their “fontanelle” (weak bones in the skull). At the root of this syndrome we find the opposition of two contrary concepts, strength/weakness. Moonlight and dew (night or early morning dew) are perceived as dangerous and especially harmful to newborns, whose skull bones have not yet joined, and are considered too fragile to withstand some natural atmospheric elements. To prevent the infant from having “broken head”, it is necessary to grease its head with “*aceite de pata*” (neatfoot oil) or with “*aceite de cusi*” (babassu oil), which is prepared and shipped by the family members who remained in Bolivia. Neatfoot oil is made from cow fat, and babassu oil is made from a type of fruit, *calucha* or babassu, which is collected from babassu palms or *Attalea speciosa* and used to treat the *cradle cap* (infantile seborrheic dermatitis) on the scalp of newborns. These are two traditional remedies used in the Eastern part of Bolivia (Amazonian region) to treat infants and newborns (Figure 1).

These remedies are usually made in Bolivia and shipped to the parents of the children

*I usually rub the fontenelle of my daughter Rosita with neatfoot oil, which is strong and warm, to prevent the coldness on her head and broken head as a result. As for the neatfoot oil, we make it boiling the cow’s paw, we put the cow´s paw to boil ... and the fat that comes out is the neatfoot oil ... my mother makes it in Bolivia and sends to me through shipment ... she sends me the neatfoot oil and the babassu oil... to protect Rosita when we go out or there is dew or moonlight ... here we use these oils less often than in Bolivia, in Bolivia we used go out more often, here we barely go out, here children are better protected.* (I 24)

The transnational family provides, through these shipments, traditional remedies with a cultural meaning that go beyond their specific function. The sending of food and medicines have a great symbolic value that strengthens the ties of the transnational family because they are related to the sphere of care^21^.

### Amartelo or sorrow disease

*The red bracelet that the girl is wearing ... is it for something? (I was referring to a very thin bracelet made of red thread that the niece of the interviewee was wearing on the right wrist…* (E)

*It’s a belief from there .... because I left my son there before departing ..., and they told me that I must wear it ... (...) The yatiri and my mother have told me. (...) ...., they say that since my son remained there, he might miss me and get sick... then, there is this red “q’aytu” (bracelet), which I make myself and it is placed around my son’s wrist to prevent him from getting sick ... because I’m not there ... when we leave our child alone ... to prevent them from getting sick ... (...) something is placed on them in order to remind them about us .... that’s what people say…*(I 14)

*Who’s taking care of your baby...?* (E)

*My mother ... I often call him and send him gifts through shipments.* (I 14)

Another culture-bound syndrome or culture-specific syndrome is the “*amartelo*”, and this health problem is caused by the distance of a loved one.

Even from a distance, mothers also take care of their children and to avoid the “*amartelo*”, they tie a thread to the children’s wrists or “*q´aytu”* (thread, in the *Quechua* language), which they had previously rubbed on their own body, the body of the person who departed. To prevent their children from missing them, mothers also create symbolic bonds of affection through phone calls and by sending them gifts^21^.

According to some authors, the discourses reporting the sorrow and the struggle for the loved ones, scenery, language, customs, etc., are a constant in the relation mental health-immigration^11^ ^,^ ^22^, which in this study were associated to the “*wayra”* and the *amartelo*. 

### Therapeutic procedures for the culture-bound syndromes

The informers describe the therapeutic procedures used in the origin (Bolivia) based on traditional or popular medicine. They consult professionals with expertise in Andean medicine (*jampiris*, *kallawayas*) to treat those diseases that doctors are unable to cure. This drastic separation between “diseases for physicians” and “diseases for traditional healers” is based on a qualitatively different conceptualization of health and disease: while indigenous medicines pursue the recovery of “human beings” in their cultural context, Biomedicine or allopathic medicine focuses on the recovery of the damaged organ. Although the health system in Bolivia tries to get adapted to include traditional medicine^23^, in Spain, the lack of professionals specialized in traditional Andean medicine defines more difuse limits for this separation and for the therapeutic procedures derived from them.

The forms of treatment of culture-bound syndromes out of their original context allow the reassessment of the reasons why we usually consider a type of therapy as effective. The ritual cures, among other reasons, are effective because: 1) they are part of the cultural scene in which the patient was socialized, 2) refer symbolic models of body perception and conception, social structure and socially shared kinship, and 3) are developed in scenarios where the protagonists of the reports of ritual healing “inhabit”. In the registered case, the Spanish healer is not part of the cultural environment in which the patient was socialized, but shares with the *jampiris* the use of the word as a basic element of the ritual healing and references of some representations proper to their original contexts (Jesus Christ, Saints and Virgin Mary). These elements seem to be sufficient for the therapy to be recognized as effective and so that in the destination countries, these immigrants continue to seek ritual healing^24^, even though it does not have the same characteristics as in their original contexts. This shows us the flexibility of our schemes in conceptualizing, explaining and treating a health condition^25^.

In the care sphere, the different ways to conceptualize health and illness among health professionals and immigrants can trigger communication problems, making the care process ineffective. The therapeutic efficacy of some of the practices described here is often questioned when they are not performed according to the rational logic of the health disciplines e are considered as irrelevant or even harmful if they remain outside the narrow margins that delimit scientific evidence. 

However, it is imperative that in the care provided to groups of different cultures, the health professional takes into account that certain health problems and the logic of the effectiveness of their treatments operate within the scope of cultural beliefs and not in that of the scientific evidence. This starting point may facilitate the intercultural dialogue and help the professional to “adapt” their care to the particularities of different cultural groups.

## Limitations

The speeches, representations and circumstances of the health sphere have been relegated to the fringe of the analytical axes and, therefore, this work requires further results that reflect the relationship between health professionals and immigrants in the contexts where the culture-bound syndromes occurr. 

Culture-bound syndromes also evolve, and globalization makes them less linked to culture and increasingly influenced by cross-border factors, although this aspect has not been evaluated in this study. 

## Conclusions

In the new environment, immigrants continue to categorize some of their illnesses as culture-bound diseases: *fright*, “*wayra*s”, *amartelo*, *pasmo de sol*, *pasmo de luna* and *pasmo de sereno*, show us that in the new environment this group maintains their worldview, on which these diseases and the therapies necessary for their cure are based.

The lack of *yatirís*, *jampiris* and *kallawayas* in the new context redistributes the relative importance of the treatments of the culture-bound syndromes, depending on the resources of the environment. The treatment for these diseases has been documented in three areas:

-In the domestic sphere (a family member takes on the role of the *yatiri* or remedies made in Bolivia are sent through shipment for the treatment of these diseases).
-In the scope of official care services (biomedicine is used to treat the biological aspect of the health problem).
-In the scope of traditional medicine (traditional healers or witch-doctors from Spain are requested).
